# HERV-W/MRSV envelope transcripts detection in blood of multiple sclerosis patients after Natalizumab treatment

**DOI:** 10.1186/1742-4690-11-S1-P131

**Published:** 2014-01-07

**Authors:** Luiz HS Nali, Guilherme S Olival, Augusto CP de Oliveira, Jorge Casseb, Jose E Vidal, Lenira Moraes, Maria CD Fink, Laura M Sumita, Camila M Romano

**Affiliations:** 1 Departamento de Moléstias Infecciosas e Parasitárias (LIMHC), Instituto de Medicina Tropical de São Paulo, Universidade de São Paulo, São Paulo, SP, Brazil; 2 Irmandade da Santa Casa de Misericórdia de São Paulo, São Paulo, SP, Brazil; 3 Instituto de Infectologia Emilio Ribas, São Paulo, SP, Brazil; 4 Laboratório de Imunodeficiências e Dermatologia, Instituto de Medicina Tropical de São Paulo e Faculdade de Medicina, Universidade de São Paulo, São Paulo, SP, Brazil; 5 Hospital das Clínicas da Faculdade de Medicina, Universidade de São Paulo, São Paulo, SP, Brazil

## 

Multiple sclerosis (MS) is an inflammatory disorder of the central nervous system (CNS) and is the most common cause of neurological disability in young adults. MS-Associated Retrovirus (MSRV) is member of Human Endogenous Retroviruses W family, and their increased activity in MS patients is associated to the disease immunopathogenesis. Natalizumab, an antibody-based therapy, hinders migration of T cells into the CNS and is currently the most potent treatment for MS. Although Natalizumab interferes with gene expression relevant for function and differentiation of lymphocytes, its effects on genes involved in immunopathogenesis are unknown. Here, we report the effect of different treatments on the HERV-W/MRSV expression in patients with relapsing-remitting MS. MRSV transcripts were quantified by qRT-PCR in peripheral blood mononuclear cells of 9 patients receiving Natalizumab for at least 6 months (MSNat group) and 11 patients under immunosuppressive treatments (MSI group). The mean age was 28 years (18-35) for MSNat group and 44 (28-54) for MSI. The mean Expanded Disability Status Scale (EDSS) score was 4 (2-6) and 2.6 (1-6.5) for MSNat and MSI groups respectively. MSRV transcripts level was slightly higher in MSNat group, although not significantly, suggesting that Natalizumab does not interfere on HERV-W expression. Patients included in Natalizumab protocol usually do not respond to other treatments and present higher EDSSs. Possibly, EDSS and age have more impact in retroelements activity, as already demonstrated. This is the first comparison of HERV/MSRV expression between different therapy groups in MS, and other studies are needed to confirm such findings.

## Funding

This work was supported by FAPESP 2010/10619-0.

